# The MEK1/2-ERK Pathway Inhibits Type I IFN Production in Plasmacytoid Dendritic Cells

**DOI:** 10.3389/fimmu.2018.00364

**Published:** 2018-02-26

**Authors:** Vaclav Janovec, Besma Aouar, Albert Font-Haro, Tomas Hofman, Katerina Trejbalova, Jan Weber, Laurence Chaperot, Joel Plumas, Daniel Olive, Patrice Dubreuil, Jacques A. Nunès, Ruzena Stranska, Ivan Hirsch

**Affiliations:** ^1^Institute of Molecular Genetics of the Czech Academy of Sciences, Prague, Czechia; ^2^Department of Genetics and Microbiology, Faculty of Sciences, Biocev, Charles University, Prague, Czechia; ^3^Institute of Organic Chemistry and Biochemistry of the Czech Academy of Sciences, Gilead Sciences & IOCB Research Centre (GSRC), Prague, Czechia; ^4^Cancer Research Center of Marseille, CNRS UMR7258, INSERM U1068, Institut Paoli-Calmettes, Aix-Marseille Université UM105, Marseille, France; ^5^Etablissement Français du Sang Rhône-Alpes, Grenoble, France; ^6^INSERM U 1209, CNRS UMR 5309, Institute for Advanced Biosciences, Université Grenoble Alpes, Grenoble, France

**Keywords:** plasmacytoid dendritic cells, toll-like receptors 7 and 9 (TLR7/9), B cell-like receptor signaling, regulatory receptors, blood dendritic cell antigen 2, MEK1/2, c-FOS, type I interferon

## Abstract

Recent studies have reported that the crosslinking of regulatory receptors (RRs), such as blood dendritic cell antigen 2 (BDCA-2) (CD303) or ILT7 (CD85g), of plasmacytoid dendritic cells (pDCs) efficiently suppresses the production of type I interferons (IFN-I, α/β/ω) and other cytokines in response to toll-like receptor 7 and 9 (TLR7/9) ligands. The exact mechanism of how this B cell receptor (BCR)-like signaling blocks TLR7/9-mediated IFN-I production is unknown. Here, we stimulated BCR-like signaling by ligation of RRs with BDCA-2 and ILT7 mAbs, hepatitis C virus particles, or BST2 expressing cells. We compared BCR-like signaling in proliferating pDC cell line GEN2.2 and in primary pDCs from healthy donors, and addressed the question of whether pharmacological targeting of BCR-like signaling can antagonize RR-induced pDC inhibition. To this end, we tested the TLR9-mediated production of IFN-I and proinflammatory cytokines in pDCs exposed to a panel of inhibitors of signaling molecules involved in BCR-like, MAPK, NF-ĸB, and calcium signaling pathways. We found that MEK1/2 inhibitors, PD0325901 and U0126 potentiated TLR9-mediated production of IFN-I in GEN2.2 cells. More importantly, MEK1/2 inhibitors significantly increased the TLR9-mediated IFN-I production blocked in both GEN2.2 cells and primary pDCs upon stimulation of BCR-like or phorbol 12-myristate 13-acetate-induced protein kinase C (PKC) signaling. Triggering of BCR-like and PKC signaling in pDCs resulted in an upregulation of the expression and phoshorylation of c-FOS, a downstream gene product of the MEK1/2-ERK pathway. We found that the total level of c-FOS was higher in proliferating GEN2.2 cells than in the resting primary pDCs. The PD0325901-facilitated restoration of the TLR9-mediated IFN-I production correlated with the abrogation of MEK1/2-ERK-c-FOS signaling. These results indicate that the MEK1/2-ERK pathway inhibits TLR9-mediated type I IFN production in pDCs and that pharmacological targeting of MEK1/2-ERK signaling could be a strategy to overcome immunotolerance of pDCs and re-establish their immunogenic activity.

## Introduction

Plasmacytoid dendritic cells (pDCs) are a highly specialized subset of dendritic cells that play a central role at the interface of innate and adaptive immunity. They are important actors in antiviral and antitumor immunity, but also potent inducers of autoimmune diseases (1–6). They sense viruses by endosomal toll-like receptors 7 and 9 (TLR7/9), recognizing ssRNA or CpG containing DNA. TLR signaling leads to the secretion of proinflammatory cytokines and chemokines, such as interleukin 1, tumor necrosis factor α (TNF-α), IL-6, IL-8, and most importantly type I IFNs (IFN-I, α/β/ω) (7–10).

In addition to TLR7/9, pDCs express multiple specific receptors that facilitate antigen capture and presentation and, moreover, regulate pDC function, preventing thus abnormal immune responses. These regulatory receptors (RRs), include Fc receptors and lectin-like receptors (11, 12), which signal through the B cell receptor (BCR)-like pathway involving spleen tyrosine kinase (SYK) associated with the immunoreceptor tyrosine-based activation motif-containing adapter of RR, Bruton’s tyrosine kinase, B-cell linker protein, phospholipase Cγ 2, MEK1/2-ERK, and induction of intracellular Ca2+ mobilization (8, 9, 12). Among these RRs, blood dendritic cell antigen 2 (BDCA-2, CD303, CLEC4C) is an lectin-like receptor (13), while immunoglobulin-like transcript (ILT7, CD85g) binds to and can be activated by bone marrow stromal cell antigen 2 (BST2, CD317, tetherin, HM1.24) protein, the expression of which is found on cells pre-exposed to IFN-I or on the surface of human cancer cells (14). Signaling *via* pDC RRs attenuates TLR-induced production of IFN-I and proinflammatory cytokines by an unknown mechanism (8–13, 15, 16). This physiological feedback mechanism of IFN control is hijacked in the pathogenesis of several chronic viral infections and cancers, leading to immune tolerance (10, 17–19). We have recently shown that hepatitis C virus (HCV) particles inhibit the production of IFN-α *via* the binding of E2 glycoprotein to RRs BDCA-2 and DCIR (dendritic cell immunoreceptor) and induce a rapid phosphorylation of AKT and ERK, in a manner similar to the cross-linking of BDCA-2 or DCIR (10, 17, 19).

Here, we addressed the question of whether specific pharmacological targeting of BCR-like signaling can restore functionality to pDCs abrogated by ligation of RRs, and what the underlying mechanism of this abrogation is. In our previous work, we demonstrated that a highly specific inhibitor of SYK blocks both BCR-like and TLR7/9 signaling and, therefore, it is not compatible with restoration of pDC function (15). In this study, we have tested the effects of inhibitors of c-Jun N-terminal kinase (JNK), MEK1/2 kinase, p38 kinase, and calcium-dependent phosphatase calcineurin, acting through a BCR-like signaling pathway, and of NF-κB activating TANK binding kinase 1 (TBK1) on the IFN-I production in pDCs exposed to a TLR9 agonist. Surprisingly, we found that inhibitors of MEK1/2 potentiated IFN-I and IL-6 production in pDC cell line GEN2.2, but not in primary pDCs stimulated by the TLR9 agonist. More importantly, inhibitors of MEK1/2 significantly increased TLR9-mediated production of IFN-I that had been blocked in both GEN2.2 cells and primary pDCs by ligation of RRs with BDCA-2 and ILT7 mAbs, or HCV particles, or with BST2 expressing cells. Moreover, the restauration of IFN-I production by MEK1/2 inhibitor was observed when TLR9 signaling had been blocked by phorbol 12-myristate 13-acetate (PMA), an agonist of protein kinase C (PKC), which stimulates MEK1/2-ERK signaling.

Furthermore, our results show that BCR-like and PKC signaling induced in pDCs the expression and phoshorylation of c-FOS, a downstream gene product of the MEK1/2-ERK pathway. c-FOS is known to associate with c-JUN to form activator protein 1 (AP-1) transcription factor and to exert within the cell a pleiotropic effect, including cell differentiation, proliferation, apoptosis, and the immune response (20–23). While a previous study reported that the c-FOS induced by tumor progression locus 2 (TPL-2) inhibits TLR9-mediated production of IFN-I in mouse macrophages and myeloid DCs, but not in pDCs (24), we show that MEK1/2-ERK-induced c-FOS was involved in the inhibition of TLR9-mediated production of IFN-I in human pDCs. Our results suggest that the MEK1/2-ERK-dependent expression and phosphorylation of c-FOS exerts an intrinsic block of TLR9-mediated production of type I IFN. Pharmacological targeting of MEK1/2-ERK signaling could be a strategy to overcome immunotolerance of pDCs and re-establish their immunogenic activity.

## Results

### MEK1/2 Inhibitor Potentiates CpG-A-Induced Production of IFN-α in pDC Cell Line GEN2.2

**Figure 1:**
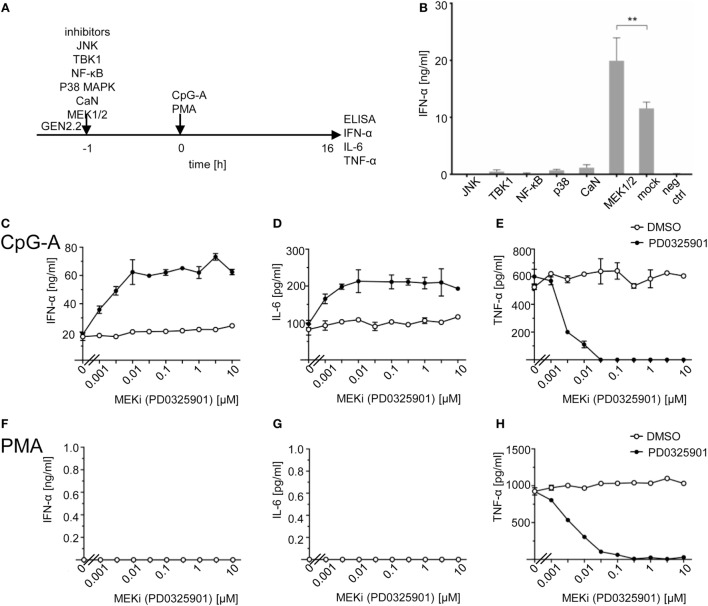
Effect of MEK1/2 inhibitor PD0325901 on cytokine production in CpG-A and phorbol myristoyl acetate (PMA)-stimulated GEN2.2 cells. **(A)** Experimental outline. GEN2.2 cells separated from MS-5 feeder cells were exposed or not to inhibitors of Jun N-terminal kinase (JNK), TANK binding kinase 1 (TBK1), NF-ĸB, p38 MAPK, calcineurin, or MEK1/2 for 1 h and then stimulated with CpG-A at 4 µg/ml. The concentration of IFN-α, IL-6, and tumor necrosis factor α (TNF-α) in the cell-free supernatant was determined by ELISA after a 16 h treatment. **(B)** The production of IFN-α by GEN2.2 cells stimulated with CpG-A in the presence of JNK (SP600125, 10 µM), TBK1 (BX795, 1 µM), NF-ĸB (Bay11-7082, 1 µM), p38 MAPK (SB253080, 1 µM), calcineurin (FK506, 0.1 µM), or MEK1/2 (PD0325901, 1 µM) inhibitors. The PD0325901 concentration-dependent production of IFN-α **(C,F)**, IL-6 **(D,G)**, and TNF-α **(E,H)** in CpG-A-induced **(C–E)** or PMA-induced **(F–H)** GEN2.2 cells. The data show mean and SEM of two independent experiments in biological triplicates **(B–H)**. **, *p* < 0.01; two-tailed Mann–Whitney test.

In order to restore TLR7/9-mediated production of IFN-I blocked by ligation of RRs, we first searched for an inhibitor of BCR signaling that does not inhibit signaling triggered by TLR7/9 agonists. To this end, we selected a panel of kinase inhibitors involved in BCR-like, MAPK, NF-ĸB, and calcium signaling, and control inhibitors of TLR7/9 signaling, and tested their effect on the production of IFN-α in a pDC cell line GEN2.2 exposed to TLR9 agonist CpG-A (Figures 1A,B; Figure S1 in Supplementary Material). To facilitate biochemical analyses of cell signaling, which is still difficult to perform in rare and *in vitro* short living human primary pDCs, we performed our studies in human pDC line GEN2.2, which shares the key features of human primary pDCs (15, 25–30).

While inhibitors of JNK (SP600125), TBK1 (BX795), NF-ĸB (Bay11-7082), p38 MAPK (SB253080), and calcineurin (FK506) inhibited dramatically IFN-α production, MEK1/2 inhibitor PD032590 significantly increased IFN-α production (*p* = 0.0022, Figure 1B). In repeated independent experiments (*N* = 34), production of IFN-α in CpG-A-stimulated GEN2.2 cells increased 2.55 ± 0.63 times (mean ± SEM, *p* < 0.0001), from 18.4 ± 1.4 ng/ml in the absence of MEK1/2 inhibitor to 44.2 ± 2.7 ng/ml in the culture pretreated with 1 µM PD0325901 (Figure S2 in Supplementary Material). In spite of the variability of IFN-α production in CpG-A-stimulated GEN2.2 cells, the ratio of IFN-α production in GEN2.2 cells cultured in the presence and in the absence of PD0325901 was highly reproducible. The same results were obtained with MEK1/2 inhibitor U0126 (data not shown). We found that in addition to IFN-α also IL-6 production in CpG-A-stimulated GEN2.2 cells was synergized by MEK1/2 inhibitor PD0325901 (Figures 1C,D), whereas production of TNF-α was inhibited (Figure 1E), suggesting that the MEK1/2-ERK pathway positively regulates TNF-α expression or secretion (31). The strongest synergistic effects on IFN-α production (synergistic index >3) were observed for combinations of ≥0.01 μM PD0325901 and 4 µg/ml CpG-A. Synergistic effects of these combinations were also demonstrated for the production of IL-6 (synergistic index >2). In contrast to the synergistic effect observed with ≥0.01 μM PD0325901, the combination of 0.001 µM PD0325901 with 4 µg/ml CpG-A had only an additive effect on the production of IL-6 (Figure 1D). In the control experiment, PMA-induced the production of TNF-α (but not that of IFN-α and IL-6), which was strongly inhibited by PD0325901 (Figures 1F–H). Collectively, these results show that the CpG-A-induced TLR9-mediated production of IFN-α and IL-6 are potentiated by MEK1/2 inhibitor PD0325901.

### MEK1/2 Inhibitor Potentiates Herpesvirus- and CpG-B-Induced Production of IFN-α in pDC Cell Line GEN2.2

**Figure 2:**
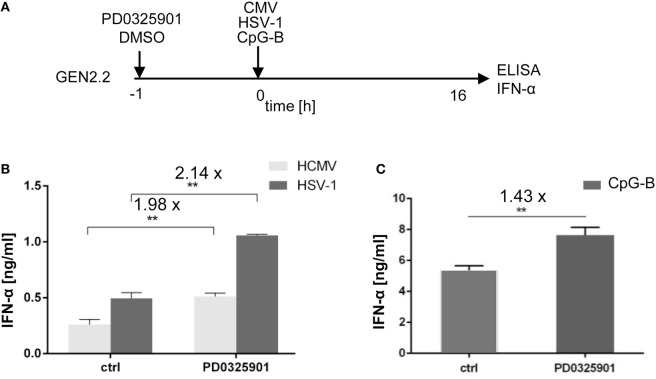
Effect of MEK1/2 inhibitor PD0325901 on the potentiation of IFN-α production stimulated with HSV-1, human cytomegalovirus (HCMV), or CpG-B. **(A)** Experimental outline. GEN2.2 cells separated from MS-5 feeder cells were incubated with the MEK1/2 inhibitor PD0325901 (1 µM) for 1 h before stimulation with HSV-1 or HCMV at the MOI of 10 TCID_50_ per cell, or with 4 µg/ml CpG-B. After a 16 h culture, the IFN-α production was determined in the cell-free supernatants by ELISA. *N* = 3, **, *p* < 0.01; two-tailed Student’s *t*-test. **(B)** The production of IFN-α by GEN2.2 cells stimulated with HSV-1 or HCMV in the presence or absence of PD0325901. **(C)** The production of IFN-α by GEN2.2 cells stimulated with CpG-B in the presence or absence of PD0325901. The data show mean and SEM of three independent experiments. *N* = 7, **, *p* < 0.01; two-tailed Mann–Whitney test.

CpG-A is a synthetic mimic of an unmethylated CpG-rich dsDNA of bacteria and viruses. Therefore, we tested whether production of IFN-α in GEN2.2 cells stimulated with natural TLR9 agonists, herpes simplex virus type 1 (HSV-1), and human cytomegalovirus (HCMV) could be potentiated with PD0325901 (Figures 2A,B). Our results show that PD0325901 significantly potentiated production of IFN-α in GEN2.2 cells exposed to HSV-1 (2.14-fold, *N* = 3, *p* = 0.0022), or HCMV (1.98-fold, *N* = 3, *p* = 0.0022).

While aggregating CpG-A is transported to the interferon-regulatory factor 7 endosomes, where activates production of IFN-I, monomeric CpG-B is transferred to the NF-κB endosomes, which leads to maturation of pDCs, formation of pro-inflammatory cytokines and only a limited production of IFN-α (7–10). PD0325901 significantly potentiated production of IFN-α in CpG-B-stimulated GEN2.2 cells (1.43-fold, *N* = 7, *p* = 0.007), although less strongly than in CpG-A-stimulated cells (Figure 2C). Taken together, MEK1/2 inhibitor PD0325901 potentiated production of IFN-α in pDC cell line GEN2.2 stimulated with synthetic TLR9 agonists CpG-A and CpG-B, and natural agonists HSV-1 and HCMV.

### MEK1/2 Inhibitors Partially Restore TLR9-Mediated IFN-α Production Blocked by Ligation of RRs with BDCA-2 and ILT7 mAbs

**Figure 3:**
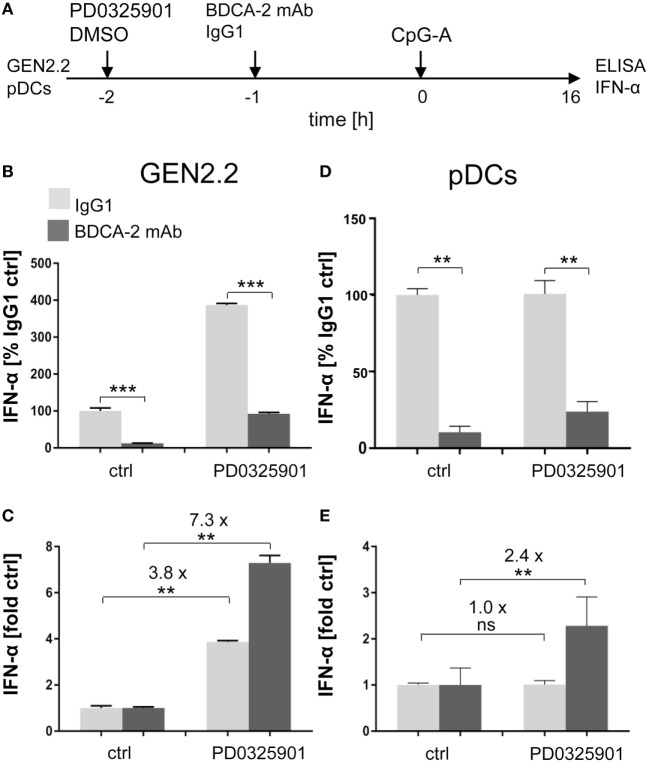
Effect of MEK1/2 inhibitor PD0325901 on the blockade of IFN-α production by ligation of regulatory receptors of GEN2.2 cells or primary plasmacytoid dendritic cells (pDCs) with blood dendritic cell antigen 2 (BDCA-2) mAb. **(A)** Experimental outline. GEN2.2 cells separated from MS-5 feeder cells or primary pDCs were incubated with the MEK1/2 inhibitor for 1 h before stimulation with BDCA-2 mAb and CpG-A. After a 16 h culture, the IFN-α production was determined in the cell-free supernatants by ELISA. **(B,D)** The IFN-α production was normalized to the level induced by CpG-A in the presence of IgG1 and in the absence of the MEK1/2 inhibitor. **(C,E)** The same data showing the IFN-α production in panels **(B–D)** were normalized to the level induced by CpG-A in the absence of the MEK1/2 inhibitor. The data show mean ± SEM of **(B,C)** six independent experiments with GEN2.2 cells, **, *p* < 0.01; ***, *p* < 0.001; two-tailed Mann–Whitney test, and **(D,E)** nine independent experiments with primary pDCs from different healthy donors, **, *p* < 0.01; two-tailed paired Wilcoxon test.

Subsequently, with respect to the ability of PD0325901 to synergize TLR7/9-mediated IFN-α production, we investigated the capacity of PD0325901 to reverse the inhibitory effect of the ligation of RRs on TLR9-mediated IFN-α production. We exposed PD0325901-pretreated GEN2.2 cells and primary pDCs to 5 µg/ml of BDCA-2 mAb and subsequently to TLR9 agonist CpG-A (Figure 3A). In the absence of the MEK1/2 inhibitor, the production of IFN-α induced in GEN2.2 cells by CpG-A was suppressed by BDCA-2 mAb to 13% (*p* = 0.0006, Figure 3B). As already shown in Figure 1C, PD0325901 significantly potentiated CpG-A-induced production of IFN-α in GEN2.2 cells (3.8-fold, *N* = 6, *p* = 0.0022, Figures 3B,C). As expected, PD0325901 potentiated the production of IFN-α inhibited in GEN2.2 cells by BDCA-2 mAb. This partial restoration of IFN-α production in GEN2.2 cells was highlighted after standardization to the quantity of IFN-α produced in the absence of PD0325901 (7.3-fold, *p* = 0.0022, Figure 3C).

As in GEN2.2 cells, exposure of primary pDCs from healthy donors to BDCA-2 mAb suppressed the production of IFN-α induced by CpG-A to 11.5% (*N* = 9, *p* = 0.0039, Figure 3D). The major difference observed in primary pDCs compared to GEN2.2 cells consisted in the lack of the potentiation of CpG-A-induced production of IFN-α by PD0325901 in the absence of BDCA-2 mAb (Figures 3B–E). In contrast, a similar restoration effect to the one in GEN2.2 was observed in primary pDCs exposed to PD0325901 prior to BDCA-2 mAb (Figures 3D,E). PD0325901 significantly restored the production of IFN-α inhibited by BDCA-2 mAb (2.4-fold, *p* = 0.0039, Figure 3E). A similar restoration effect was observed with PD0325901 at 10 nM concentration (Figure S3 in Supplementary Material) and with MEK1/2 inhibitor U0126 using ILT7 mAb for crosslinking RR (Figure S4 in Supplementary Material). In conclusion, these results show that MEK1/2 inhibitors significantly increased the TLR9-mediated IFN-I production blocked by ligation of RRs.

### MEK1/2 Inhibitor Restores TLR7/9-Mediated IFN-α Production Blocked by HCV Virions

**Figure 4:**
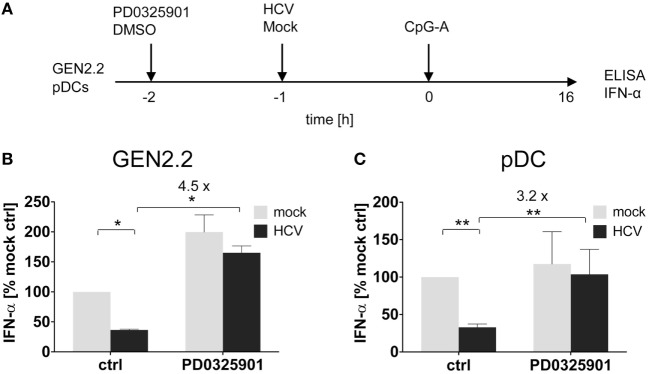
Effect of MEK1/2 inhibition on the hepatitis C virus (HCV) blockade of IFN-α in GEN2.2 cells or primary plasmacytoid dendritic cells (pDCs). **(A)** Experimental outline. GEN2.2 cells separated from MS-5 feeder cells **(B)**, or primary pDCs **(C)**, were incubated with 1 µM MEK1/2 inhibitor PD0325901 for 1 h and then treated with HCV virions at MOI = 10 geq/cell for 1 h before CpG-A stimulation. After a 16 h culture, the IFN-α production was determined in the cell-free supernatants by ELISA. **(B,C)** The IFN-α production was normalized to the level induced by CpG-A in the presence of a mock-infected control and in the absence of PD0325901. The data show mean ± SEM of **(B)** two independent experiments with GEN2.2 cells, *, *p* < 0.05; unpaired, two-tailed *t*-test and **(C)** ten independent experiments with primary pDCs from different healthy donors, **, *p* < 0.01; two-tailed paired Wilcoxon test.

We and others reported that some viruses, such as HCV (19, 32), HBV (18), or HIV (17), interact *via* their envelope glycoproteins with RR BDCA-2 expressed on pDCs, and activate the BCR-like pathway leading to the inhibition of IFN-α production. We tested whether MEK1/2 inhibitor PD0325901 restores IFN-α production in pDC cell line GEN2.2 (Figures 4A,B) and in primary pDCs (Figures 4A,C) stimulated with CpG-A, and in parallel exposed to HCV particles (10 HCV geq/cell). We confirmed that in the absence of MEK1/2 inhibitor, HCV virions inhibited IFN-α production in both cell types, to 35% in GEN2.2 cells (Figure 4B) and to 34% in primary pDCs (Figure 4C) (19, 33). We observed that the treatment with PD0325901 significantly restored CpG-A-stimulated production of IFN-α inhibited by HCV virions in GEN2.2 cells (4.2-fold, *p* = 0.025, Figure 4B) and in primary pDCs (3.2-fold, *p* = 0.0059, Figure 4C), in a more robust way than that observed with BDCA-2 mAb (Figure 3). Collectively, pharmacological targeting of MEK1/2-ERK abrogates the HCV suppression of IFN-α production.

### MEK1/2 Inhibitor Restores TLR9-Mediated IFN-α Production Blocked by Ligation of RRs with BST2 Expressing HEK293T Cells

**Figure 5:**
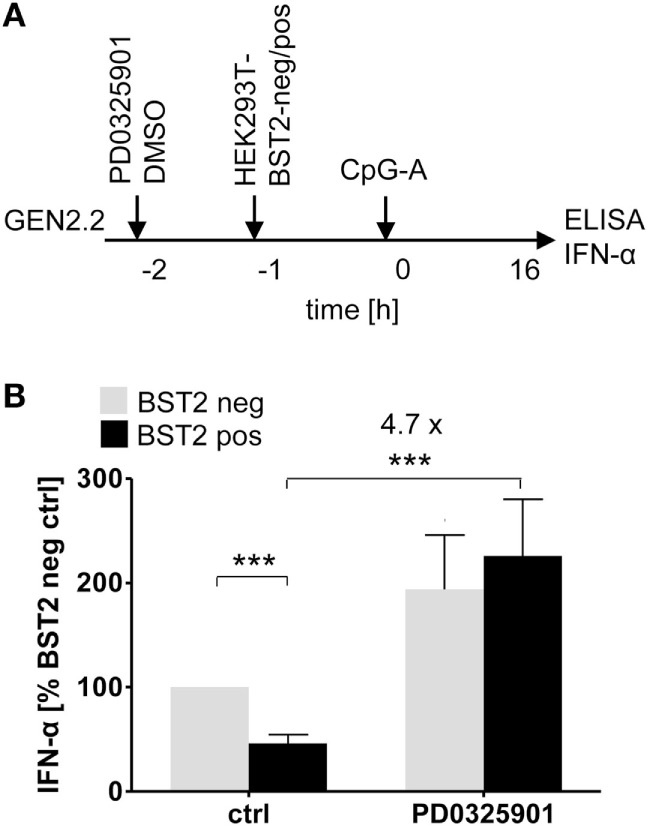
Effect of MEK1/2 inhibition on the blockade of IFN-α by co-culture of GEN2.2 cells with BST2-expressing HEK293T cells. **(A)** Experimental outline. In total 10^5^ GEN2.2 cells pretreated with 1 µM PD0325901 were added to a monolayer of 10^5^ control HEK293T cells or to the same amount of BST2-expressing HEK293T cells in a volume of 200 µl. The proportion of BST2-expressing cells in the lentivirus-transduced HEK293T cells was determined by flow cytometry using the anti-BST2-PE antibody (Figure S4 in Supplementary Material). The co-cultures of GEN2.2 and HEK293T cells were kept for 1 h at 37°C before adding CpG-A. After a 16 h culture, the IFN-α production was determined in the cell-free supernatants by ELISA. **(B)** The IFN-α production was normalized to the IFN-α level induced in GEN2.2 cells by CpG-A in co-culture with the mock-transduced BST2-negative HEK293T cells and in the absence of PD0325901. The data show mean ± SEM of five independent co-culture experiments of GEN2.2 cells with BST2-negative or BST2-positive HEK293 cells, *, *p* < 0.05; ***, *p* < 0.001; two-tailed Mann–Whitney test.

ILT7 is another pDC-specific receptor with a regulatory function that signals through the BCR-like pathway and inhibits TLR-mediated IFN-α production (11). In order to evaluate the restoration effect of MEK1/2 inhibitors, we exposed GEN2.2 cells to a HEK293T cell line which expressed BST2, a natural ligand of ILT7 (11), in approximately 95% of cells (Figure 5A; Figure S5 in Supplementary Material). In the absence of MEK1/2 inhibitor, the co-culture of GEN2.2 cells with the BST2 expressing HEK293T inhibited IFN-α production induced by CpG-A to 47.4% (*p* = 0.001, Figure 5B). When the GEN2.2 cells were exposed to 1 µM PD0325901 prior to co-culture with BST2 expressing HEK293T cells and CpG-A simulation, the IFN-α production significantly increased (4.7-fold, *p* = 0.001, Figure 5B). In conclusion, the MEK1/2 inhibitor restored TLR9-mediated IFN-α production blocked by ligation of RR ILT7 with BST2.

### MEK1/2 Inhibitor Restores TLR9-Mediated IFN-α Production Blocked by PMA

**Figure 6:**
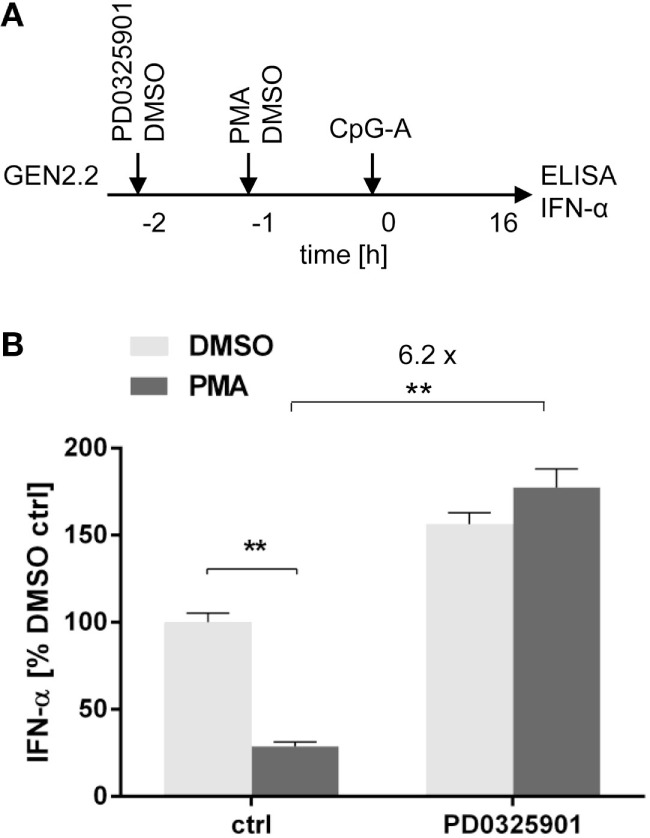
Effect of MEK1/2 inhibitor PD0325901 on the blockade of IFN-α production in phorbol myristoyl acetate (PMA)-stimulated GEN2.2 cells. **(A)** Experimental outline. GEN2.2 cells separated from MS-5 feeder cells were incubated with the MEK1/2 inhibitor for 1 h before stimulation with PMA. After a 16 h culture, the IFN-α production was determined in the cell-free supernatants by ELISA. **(B)** The IFN-α production was normalized to the level induced by CpG-A in the presence of DMSO and in the absence of the MEK1/2 inhibitor. The data show mean ± SEM of six independent experiments with GEN2.2 cells. **, *p* < 0.01; two-tailed Mann–Whitney test.

A recent study showed that treatment of pDCs with PMA, an agonist of PKC activating MEK1/2-ERK signaling pathway, has led to a dose-dependent reduction of IFN-α secretion (34). We investigated the capacity of PD0325901 to reverse the inhibitory effect of PMA on TLR9-mediated IFN-α production (Figure 6A). In the absence of the MEK1/2 inhibitor, the production of IFN-α induced in GEN2.2 cells by CpG-A was suppressed by PMA to 25% (*N* = 6, *p* = 0.0022, Figure 6B). PD0325901 significantly potentiated CpG-A-induced production of IFN-α in GEN2.2 cells (1.56-fold, *N* = 6, *p* = 0.0022, Figure 6B). PD0325901 completely restored the production of IFN-α inhibited in GEN2.2 cells by PMA (6.18-fold, *p* = 0.0022, Figure 6B). In conclusion, activation of MEK1/2-ERK pathway by PMA inhibited the TLR9-mediated IFN-α production and this effect was abrogated by PD0325901.

### c-FOS Levels in pDC Cell Line GEN2.2 Are Higher Than Those in Primary pDCs

**Figure 7:**
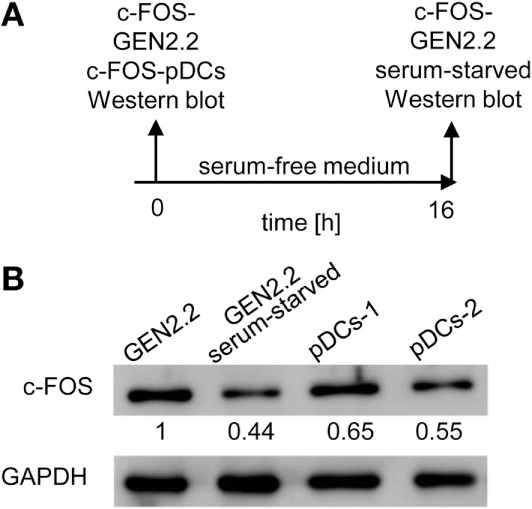
c-FOS in proliferating GEN2.2 cells, GEN2.2 cells starved in the serum-free medium and in primary plasmacytoid dendritic cells (pDCs). **(A)** Experimental outline. Total cell extracts were prepared from GEN2.2 cells immediately after their separation from MS-5 feeder cells (GEN2.2), GEN2.2 cells separated from MS-5 feeder cells and starved overnight in the serum-free medium (GEN2.2 serum-starved), and from primary pDCs isolated from two healthy donors by magnetic-bead purification without any further culture (pDCs-1, pDCs-2). **(B)** c-FOS levels were determined in the total cell extract by Western blotting by rabbit polyclonal Ab c-FOS (sc-52). The values shown below each band represent relative quantity of c-FOS determined by densitometry normalized to proliferating GEN2.2 cells. GAPDH was used as a loading control.

The implication of MEK1/2 in the crosstalk of BCR-like and TLR7/9 signaling led us to investigate the role of c-FOS, a downstream immediate early response gene (20), in the regulation of TLR7/9 response. To this end, we compared the levels of c-FOS protein in the GEN2.2 cell line with those in primary pDCs (Figures 7A,B). We found that the quantity of c-FOS in GEN2.2 cells cultured in complete medium was approximately double that of primary pDCs (Figure 7B). Among numerous transcription factor binding sites in the upstream promoter region of *c-FOS*, the serum response element plays a central regulatory role in responding to external stimuli by growth factors and mitogens (20). To assess the basal level of c-FOS in GEN2.2 cells, we determined c-FOS levels in GEN2.2 starved for 16 h in serum-free medium. The starvation reduced the quantity of c-FOS in GEN2.2 cells to the level present in primary pDCs (Figure 7B).

### Expression of c-FOS Induced by BDCA-2 Crosslinking Precedes and Exceeds That Induced by CpG-A

**Figure 8:**
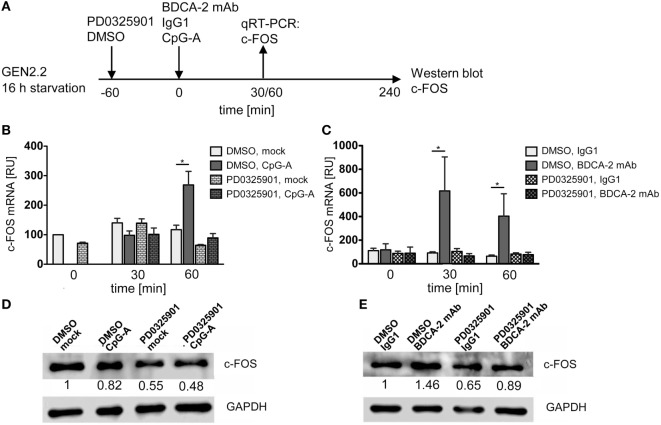
*c-FOS* mRNA and protein expression in GEN2.2 stimulated with TLR9 or RR agonists. **(A)** Experimental outline. GEN2.2 cells separated from MS-5 feeder cells and starved in a serum-free medium for 16 h were pretreated or not with MEK1/2 inhibitor PD0325901 for 1 h and then exposed to CpG-A **(B,D)** or blood dendritic cell antigen 2 (BDCA-2) mAb **(C,E)**. **(B,C)** The expression of human *c-FOS* mRNA was quantified after 30 or 60-min exposure to CpG-A **(B)** or BDCA-2 mAb **(C)** by TaqMan qRT-PCR in the total cellular RNA. The data normalized to time zero show mean ± SEM of three independent experiments; *, *p* < 0.05; two-tailed Mann–Whitney test. **(D,E)** c-FOS protein levels were determined after 240 min exposure to CpG-A **(D)** or BDCA-2 mAb **(E)** in the total cell extract by Western blotting by rabbit polyclonal Ab c-FOS (sc-52). Relative quantity of c-FOS protein normalized to mock-treated GEN2.2 cells determined by densitometry is shown below each band. GAPDH was used as a loading control (representative result of three independent experiments).

We determined the effect of CpG-A and BDCA-2 mAb on the kinetics of expression of the *c-FOS* gene in the serum-starved GEN2.2 cells pretreated or not with PD0325901 (Figure 8A). The peak of *c-FOS* transcription occurred 60 min after stimulation with CpG-A (Figure 8B), while crosslinking of BDCA-2 induced an earlier (30 min) and a stronger transcription of *c-FOS* (Figure 8C). Pretreatment with PD0325901 blocked the induction of *c-FOS* transcription by both CpG-A and BDCA-2 mAb (Figures 8B,C). In addition to the quantification of *c-FOS* mRNA by qRT-PCR, we determined the c-FOS protein levels in the serum-starved GEN2.2 cells exposed to CpG-A or BDCA-2 mAb by western blot (Figures 8D,E). While stimulation of GEN2.2 cells with CpG-A decreased the level of c-FOS protein (0.82-fold), crosslinking of BDCA-2 increased the production of c-FOS (1.46-fold).

### PD0325901 Inhibits G1/S Phase Transition of GEN2.2 Cell Cycle

**Figure 9:**
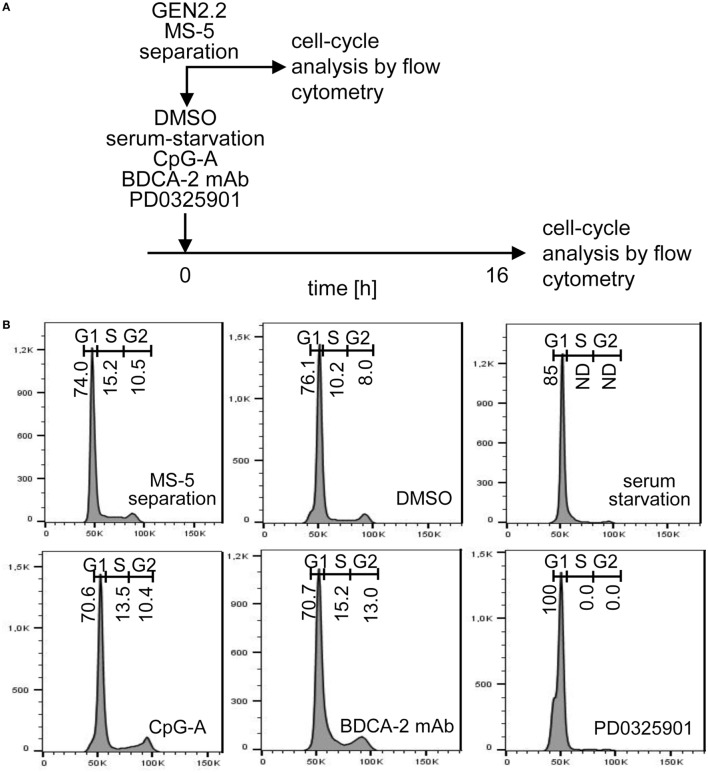
Cell cycle analysis of GEN2.2 cells cultured in the presence of PD0325901, CpG-A, blood dendritic cell antigen 2 (BDCA-2) mAb, or in serum-free medium. **(A)** Experimental outline. GEN2.2 cells separated from MS-5 feeder cells were exposed for 16 hr to 1 µM PD0325901, 4 µg/ml CpG-A, 0.25 µg/ml BDCA-2 mAb, or DMSO (mock-treated control), or analyzed immediately after separation from MS-5 cells. **(B)** Cell cycle analysis using Hoechst 33342 stain. Histograms of live GEN2.2 cells (representative result of three independent experiments) stained with Hoechst 33342 dye showing DNA content distribution. Live/Dead cell discrimination was performed by Zombie Green™ Fixable Viability Kit. ND, not determined.

While pDC line GEN2.2 shares many features with primary pDCs (15, 25–30), GEN2.2 cells principally differ from primary pDCs by their capacity to proliferate. To further analyze this difference, we tested whether the higher basal level of c-FOS in proliferating GEN2.2 cells relative to primary pDCs is related to the MEK1/2-ERK-mediated c-FOS induction and G1/S phase transition of the cell cycle (21) (Figures 9A,B). Proliferating GEN2.2 cells were treated with PD0325901, corresponding concentration of DMSO, CpG-A, and BDCA-2 mAb, or starved in serum-free medium, and the impact on their cell cycle was analyzed 16 h later. Cell cycle of a control culture of GEN2.2 cells was analyzed immediately after separation from MS-5 cells. We found that the MEK1/2-ERK pathway inhibitor PD0325901 blocked the cell cycle in proliferating GEN2.2 cells. The cell cycle was also strongly inhibited in the serum-starved GEN2.2 cells, although the impairment of the cell cycle in this cell culture did not permit to calculate residual S phase and G2/M phase cells according to mathematical model used in our analyses. As expected, BDCA-2 crosslinking did not block, but stimulated G1/S phase transition, consistently with increase of c-FOS level in BDCA-2-crosslinked cells (Figure 8E). CpG-A stimulation had only slight effect on G1/S phase transition. Cell cycle arrest in the GEN2.2 cells pretreated with PD0325901 or starved for serum (Figure 9B) correlated with the decline in the c-FOS level (Figures 7 and 8D,E) and with the potentiation of CpG-A-induced production of IFN-α (Figure 1C).

### BDCA-2 Crosslinking Induces Phosphorylation of c-FOS

**Figure 10:**
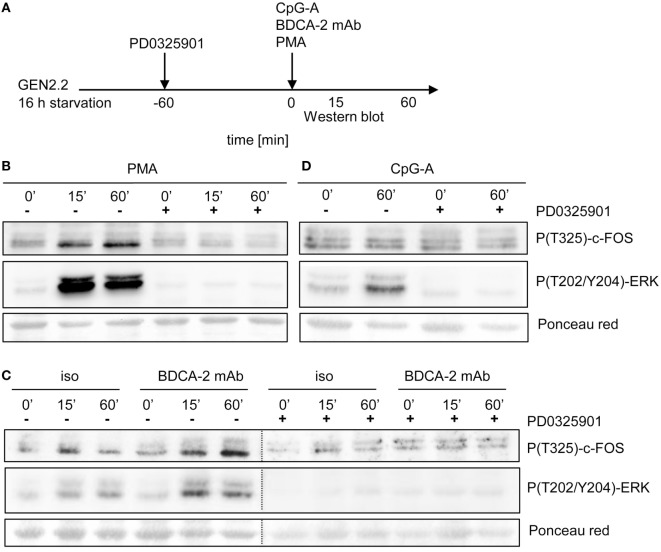
Activation of c-FOS and ERK in GEN2.2 cells stimulated with phorbol myristoyl acetate (PMA), blood dendritic cell antigen 2 (BDCA-2) mAb and CpG-A. **(A)** Experimental outline. GEN2.2 cells separated from MS-5 feeder cells and starved in a serum-free medium for 16 h were pretreated or not with MEK1/2 inhibitor PD0325901 for 1 h and then stimulated with PMA **(B)**, BDCA-2 mAb **(C)**, or CpG-A **(D)**. The activation of c-FOS was evaluated by analysis of c-FOS phosphorylation using Western blotting with the P(T325)-c-FOS antibody. The phosphorylation of ERK-1 was determined by P(T202/Y204) ERK-1. Ponceau red was used as a loading control. Figure **(C)** is composed of two images of two different gels with samples from the same experiment. The two images are separated by a dotted line. Full scans of the original gels are shown in Figure S7 in Supplementary Material.

It was reported that ERK1/2-mediated post-translational phosphorylation enhances c-FOS stability and transcriptional activity (20, 22, 23). We assessed the phosphorylation of ERK1/2 at T202/Y204 and c-FOS at T325 in serum-starved GEN2.2 cells treated with RR agonist BDCA-2 mAb, TLR9 agonist CpG-A, and PKC agonist PMA (Figure 10A). c-FOS phosphorylation was analyzed using Western blotting with the P(T325)-c-FOS antibody. In the control experiment, 15 or 60 min exposure of GEN2.2 cells to PMA-induced strong phosphorylation of ERK1/2 at T202/Y204 and the c-FOS at T325, which was efficiently inhibited by PD0325901 (Figure 10B). The levels of total c-FOS and ERK1/2 remained unchanged in GEN2.2 cells stimulated with PMA for 15 or 60 min (Figure S6 in Supplementary Material). Stimulation with BDCA-2 mAb induced strong phosphorylation of ERK1/2 at T202/Y204 and the c-FOS phosphorylation at T325, which was abrogated by pretreatment with MEK1/2 inhibitor PD0325901 (Figure 10C; Figure S7 in Supplementary Material). In contrast to BDCA-2 mAb or PMA, CpG-A-induced ERK-1/2 T202/Y204 phosphorylation without inducing the phosphorylation of c-FOS T325 (Figure 10D). In conclusion, all three agonists induced phosphorylation of ERK-1/2, which was inhibited by 1 µM PD0325901. BDCA-2 mAb and PMA induced phosphorylation of c-FOS while CpG-A did not. The phosphorylation of c-FOS was inhibited by PD0325901, which is consistent with the regulation of c-FOS by MEK1/2-ERK signaling.

### BDCA2 Crosslinking in GEN2.2 Cells and Primary pDCs Induces Upregulation of c-FOS

**Figure 11:**
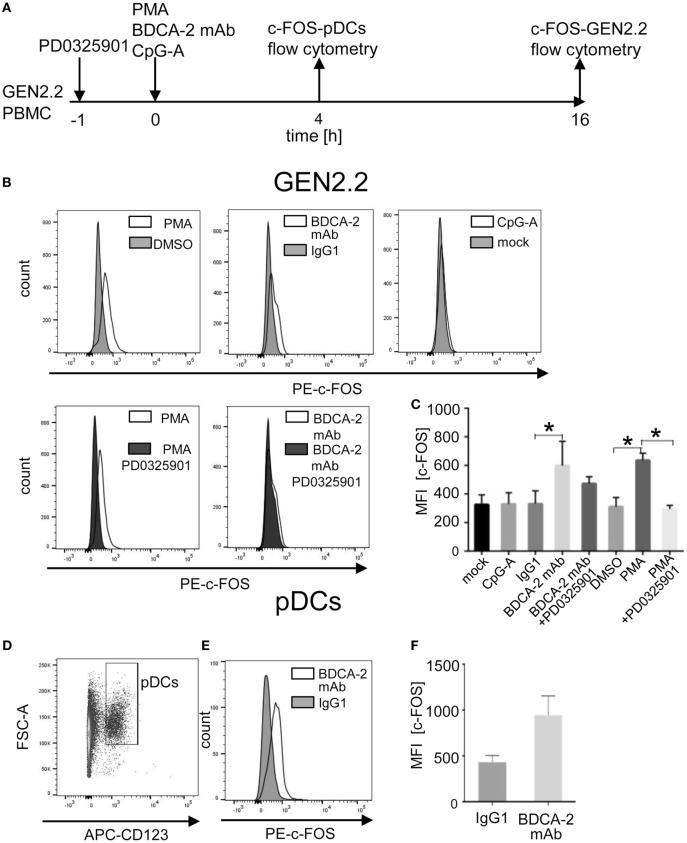
Induction of total c-FOS in GEN2.2 cells and primary plasmacytoid dendritic cells (pDCs). **(A)** Experimental outline. GEN2.2 cells separated from MS-5 feeder cells exposed or not to PD0325901 were stimulated with phorbol myristoyl acetate (PMA), blood dendritic cell antigen 2 (BDCA-2) mAb, or CpG-A for 16 h. Peripheral blood mononuclear cells (PBMCs) of healthy donors were exposed to BDCA-2 mAb or IgG1 isotype Ab and quantity of the total c-FOS in pDCs gated from PBMCs was determined 4 h later. **(B)** Fluorescence intensity of the total c-FOS in GEN2.2 cells. Viable GEN2.2 cells were gated according to Live/Dead Zombie Green kit, semipermeabilized and stained with PE-conjugated c-FOS (9F6) rabbit mAb. Control light shaded areas show c-FOS in unstimulated PD0325901 mock-treated GEN2.2 cells. Dark shaded areas show c-FOS in PD0325901-treated GEN2.2 cells. Representative result of three independent experiments. **(C)** The data show mean MFI ± SEM of the total c-FOS in GEN2.2 cells determined in three independent experiments. *, *p* < 0.05; unpaired, two-tailed *t*-test. **(D)** Primary pDCs in PBMCs were gated negatively for Zombie green- (living cells) and FITC-Lin^−^ and positively for APC-CD123+. **(E)** c-FOS in semipermeabilized primary pDCs gated from PBMCs stimulated with BDCA-2 mAb or IgG1 isotype for 4 h was stained with PE-c-FOS (9F6) rabbit mAb. Representative result of three independent experiments with PBMCs from different healthy donors. **(F)** The data show mean MFI ± SEM of the total c-FOS in primary pDCs gated from BDCA-2-stimulated or unstimulated PBMCs determined in three independent experiments in PBMCs of different donors.

A recent study reported that BDCA-2 crosslinking and internalization result in up to 16 hr-lasting resistance of pDCs to TLR7/9-mediated stimulation suggesting a stability of the IFN-I inhibitory signal (35). Although c-FOS expression is usually rapid and transient, c-FOS stability is enhanced by phosphorylation (20, 22, 23). These observations led us to investigate the stability of c-FOS levels after stimulation of the BCR-like or TLR9 pathways. We analysed the quantity of c-FOS in the GEN2.2 cell line 16 h after stimulation with the control PMA, BDCA-2 mAb, and CpG-A by flow cytometry in the presence or absence of PD0325901 (Figure 11A). The results show that stimulation with PMA and BDCA-2 mAb induced a sustained increase in c-FOS levels, while stimulation with CpG-A did not (Figure 11B). The increase in c-FOS levels in the PMA and BDCA-2 stimulated GEN2.2 cells was inhibited by PD0325901. MFI of c-FOS significantly increased after BDCA-2 crosslinking and PMA stimulation of GEN2.2 cells but not after stimulation with CpG-A (*N* = 3, Figure 11C). While PD0325901 almost completely inhibited c-FOS production in GEN2.2 cells stimulated by PMA, it exerted only partial inhibition in BDCA-2 mAb-crosslinked cells.

To assess whether stimulation of BDCA-2 in primary pDCs also upregulates the expression of c-FOS, we exposed PBMCs from three healthy donors to BDCA-2 mAb and determined the level of c-FOS in a rapidly dying population of primary pDCs 4 hr later. Because the low proportion of pDCs in PBMCs makes their biochemical analyses difficult, we used flow cytometry for this purpose (Figures 11A, D–F). The MFI of c-FOS induced by BDCA-2 mAb increased 2.19 ± 0.85 times compared to isotypic IgG1 control in pDCs (Figure 11E). These results show that the stimulation of RRs of pDCs results in a sustained increase of the c-FOS level not only in the GEN2.2 cell line but also in primary pDCs.

## Discussion

Our results demonstrate the important role of MEK1/2-ERK signaling in the RR-mediated inhibition of IFN-α and IL-6 production in pDCs. We showed that MEK1/2 inhibitors PD0325901 and U0126 were the only constituents of the panel of inhibitors of BCR-like signaling that not only did not abrogate, but even stimulated TLR9 signaling in GEN2.2 cells. Pharmacological targeting of MEK1/2 in GEN2.2 cells or primary pDCs significantly abrogated inhibition of the TLR9-mediated production of IFN-I induced by BCR-like or PKC signaling. Both BCR-like and PKC signaling activated MEK1/2-ERK pathway.

The molecular mechanism by which the ligation of the RRs antagonizes TLR7/9 signaling in pDCs remains elusive despite years of intense research in many laboratories (8–10, 12–14, 16, 35). We show here that MEK1/2-ERK signaling upregulated the production and phosphorylation of c-FOS. Thus, the potentiation of IFN-I by PD0325901 treatment of GEN2.2 cells could be consequence of a natural role of c-FOS in cell proliferation. The role of c-FOS in the activation of the G1/S cell cycle transition and in the inhibition of IFN-α and IL-6 production in GEN2.2 cells should be further investigated. A higher level of c-FOS in proliferating GEN2.2 cells in comparison with resting primary pDCs represents a major difference between these cell types and is consistent with the different outcome of MEK1/2-ERK inhibition. The demonstration of the synergistic effect of MEK1/2 inhibitors on the CpG-A-induced production of IFN-α suggests that under steady-state conditions a natural intrinsic block regulated by MEK1/2 controls the IFN-α level in GEN2.2 cells to a higher level than that in primary pDCs. Release of this block could be a part of the restoration mechanism of IFN-α by MEK1/2 inhibitors in pDCs exposed to RR agonists.

The levels of inhibition of IFN-I production by crosslinking of RR and their restoration by MEK1/2-ERK inhibitors varied depending on the RR ligand. This could be related to differences in the cell-surface distribution of targeted receptors (BDCA-2, ILT7, DCIR) and avidity of tested ligands (BDCA-2 and ILT7 mAbs, HCV particles, or BST2 expressing cells). Among them, BDCA-2 mAb was the most potent inhibitor of IFN-I production. Surprisingly the relative levels of inhibition and restoration of IFN-I production were similar in GEN2.2 cell line and primary pDCs. In addition to differences in receptor/ligand interactions, the levels of inhibition and restoration of IFN-I production were dependent on the mechanism of stimulation of MEK1/2-ERK pathway by BCR-like or PKC signaling. While pretreatment with PD0325901 led to almost complete inhibition of c-FOS expression induced by PMA, c-FOS expression induced by BDCA-2 mAb was only partially inhibited. This suggests that expression of c-FOS induced by BDCA-2 crosslinking and internalization could be partially MEK1/2-ERK independent.

MEK1/2 inhibitor PD0325901 potentiated production of IFN-α in pDC cell line GEN2.2 stimulated by both synthetic (CpG-A and CpG-B) and natural (HSV-1 and HCMV) agonists. In the absence of PD0325901, exposure of pDCs to HSV-1 and HCMV results in a non-permissive infection and TLR9-mediated production of IFN-α (36, 37). Interestingly, the quantity of IFN-α produced by murine pDCs exposed to murine CMV (MCMV) is down-modulated by MCMV-induced stimulation of DAP12, an adaptor molecule of murine RR (38). Recent study demonstrated that EBV and double-stranded DNA viruses induce TRIM29 leading to suppression of IFN-α production (39). The potential role of TRIM29 in HSV-1 and HCVM-mediated inhibition of IFN-α production in pDCs needs to be clarified.

A previous report implicated c-FOS induced by MAP3-kinase TPL-2 in the negative regulation of TLR9-mediated production of IFN-β in mouse macrophages and myeloid (mDCs), but not in mouse pDCs (24). In contrast, we show here that c-FOS induced by MEK1/2-ERK signaling is involved in the regulation of TLR9 signaling in human pDCs. It is possible that TPL-2 and MEK1/2-ERK signaling are interpreted differently in mouse and human pDCs compared with macrophages and mDCs as a consequence of an interaction of ERK activation with other signaling pathways triggered by TLR9 (18). Several cell type-specific studies have shown that the interaction of TLR7/9 with BCR-like signaling may be regulated in a different way in human pDCs (7, 12, 14, 16, 35, 40).

Activation of Ras/MEK1/2/ERK downregulates expression of IFN-I also in human epithelial cancer cells (41). Together with our experiments, these results suggest that MEK1/2-ERK signaling can play a general role in regulation of IFN-I. Another recent study demonstrated that MEK1/2-ERK-mediated phosphorylation of c-FOS in HCV-infected hepatocytes induced miR-21, which targeted MyD88 and IRAK1 and contributed to the suppression of IFN-I production (42). We did not detect a significant increase of miR-21 level in GEN2.2 cells exposed to BDCA-2 mAb or CpG-A (not shown).

We have demonstrated that inhibitors of MEK1/2 restore the production of IFN-I inhibited by ligation of RRs with HCV particles or with BST2 expressing cancer cells. These results suggest that pharmacological targeting of MEK1/2-ERK signaling could be a strategy to overcome immunotolerance of pDCs and re-establish their immunogenic activity. This finding complements our previous results showing that an inhibitor of SYK, a protein kinase involved in both TLR7/9 and BCR-like pathways, could be a useful tool to suppress the overproduction of IFN-I and to re-establish tolerogenic homeostatic functions of pDCs (15). The role of IFN-I in the pathogenesis of chronic viral infections and cancer is unclear and ambivalent. IFN-I responses are critical in the early phases of immune response to infections, but the chronic and systemic activation of pDCs can paradoxically lead to deleterious consequences for the immune system (43, 44). It is likely that an intense signaling occurs in the mucosa, involving a local accumulation of pDCs producing IFN-I early during HIV-1 infection, which is associated with the chronic activation of the immune system (45, 46). While in this era of great success of direct-acting antivirals against HIV and HCV the stimulation of IFN response might represent an adjuvant therapy, important namely in the case of virus escape, the induction of IFN-I in combination with existing antivirals may cure HBV infection (47–49). IFN-I also plays an important role in antitumor immunity (3, 50). The addition of exogenous IFN-α reverts the immunotolerance of tumor-associated pDCs in breast and ovarian carcinoma (4, 51). Pharmacological targeting of MEK1/2 signaling may constitute an attractive new approach to study mechanisms of modulation of pDC activation in pathophysiological conditions such as chronic viral infections and cancer.

## Materials and Methods

### Isolation and Culture of Primary pDCs

Peripheral blood mononuclear cells (PBMCs) from healthy anonymous donors were obtained from the national blood services (Etablissement Francais du Sang, Marseille, France). Blood samples were obtained after written consent following the approval of the EFS, Marseille, France, and the Center de Recherche en Cancérologie de Marseille (CRCM) in accordance to the convention signed the 20th May 2014. pDCs purified from PBMCs as described previously were 75–95% pure, with a contamination of less than 5% mDCs (32, 33, 52, 53). Isolated pDCs were cultured in RPMI 1640 supplemented with 10% fetal calf serum (FCS). To optimize viability in overnight experiments, recombinant IL-3 (R&D Systems Europe, Ltd., Abingdon, UK) was added to a final concentration of 10 ng/mL.

### pDC Line GEN2.2

Human pDC line GEN2.2 (25) was grown in a RMPI 1640 medium supplemented with L-glutamine, 10% FCS, 1% sodium pyruvate, and 1% MEM nonessential amino acids, on a monolayer of the murine stromal feeder cell line MS-5 grown in RPMI 1640 supplemented with L-glutamine, 10% FCS, and 1% sodium pyruvate. For the measurement of cytokine production, the dynamic flow cytometry and the Western blot experiments, GEN2.2 cells were separated from the MS-5 feeder cells.

### Inhibitors, Antibodies, and Reagents

MEK-1/2 inhibitor PD0325901 obtained from InvivoGen (Toulouse, France) and U0126 obtained from Sigma (Sigma-Aldrich, Lyon, France) were used as recommended by supplier. PD0325901 is a selective non-ATP-competitive allosteric MEK1/2 inhibitor with *in vitro* IC50 0.33 nM, which was shown to be specific against a panel of 70 different kinases at 10 µM range (54). U0126 inhibits MEK 1/2 with an *in vitro* IC50 of 0.5 µM. JNK inhibitor SP600125, TBK1 inhibitor BX795, NF-ĸB inhibitor Bay11-7082, p38 MAPK inhibitor SB253080, and calcineurin inhibitor FK506 were all purchased from InvivoGen, San Diego, USA. For *in vitro* pDC stimulation assays, CpG-A (ODN 2216), CpG-B (ODN 2006), and PMA (all InvivoGen, San Diego, USA), and BDCA-2 antibody (Miltenyi Biotech, Paris, France), and ILT7 antibody (eBioscience) were used.

### *In Vitro* pDC Stimulation

To determine cytokine production, purified primary human pDCs (in the presence of IL-3) or GEN2.2 cells were kept at a concentration of 10^6^ cells/ml aliquoted in 100 µl quantities in 96-well round-bottom culture plates and stimulated with 4 µg/ml CpG-A or CpG-B, 25 ng/ml PMA, 20 µg/ml of BDCA-2 or ILT7 antibody, or 10 HCV geq/cell for 16 h. In some experiments, BDCA-2 or ILT7 antibody-exposed cells were further crosslinked with goat-antimouse F(ab′)_2_ (15 µg/ml) (Jackson ImmunoResearch).

### Production and Purification of Cell Culture-Derived HCVcc (JFH-1 3 M), HSV-1, and HCMV Virus Stocks

Hepatitis C virus cc particles were prepared in Huh7.5 cells (55) (kindly provided by APATH L.L.C.) on the basis of plasmid pJFH-1 displaying mutations, F172C and P173S in core and N534K in E2 (56), as described previously (33). The ultracentrifuged virus purified through a cushion of 20% sucrose was resuspended in RPMI 1640 to obtain a 1,000-fold concentrated virus suspension containing 10^7^ FFU_Huh7.5_/10^11^ HCV RNA copies/ml. Stocks of HSV-1, strain Praha, and HCMV, strain AD-169, were prepared as described previously (57, 58).

### Preparation of BST2 Expressing HEK293T Cells

The BST2 sequence from pCMV-Sport6-BST2 was cloned into the pRRL.PPT.SF.i2GFPp expression vector to produce a lentiviral vector pRRL-BST2-GFP. HEK293T cells were transduced by the resulting lentivirus construct at MOI = 10 and GFP-positive cells were selected by FACSAria (BD Biosciences). The expression of GFP and BST2 in transduced cells was determined by flow cytometry by LSRII (BD Biosciences).

### Determination of *c-FOS* Expression

Total cellular RNA was isolated using RNeasy Mini Kit (Qiagen). cDNA was synthesized using High Capacity cDNA Reverse Transcription Kit (Applied Biosystems). Human *c-FOS* was amplified with SYBR^®^ Green PCR Master Mix (Applied Biosystems) using the following primers: *c-FOS*: forward: 5′-CAAGCGGAGACAGAC CAACT-3′and reverse 5′-AGTCAGATCAAGGGAAGCCA-3′; GAPDH: forward: 5′-GCGAGATCCCTCCAAAATCAA-3′and reverse 5′-GTTCACACCCATGACGAACAT-3′. Relative expression levels were calculated using 2^−ΔΔCT^ method. *GAPDH* was used as endogenous control.

### Determination of ERK and c-FOS by Immunobloting

Total c-FOS and ERK in the whole cell lysate of GEN2.2 cells or primary pDCs were determined by Western blotting by means of rabbit polyclonal c-FOS (sc-52) and ERK1/2 (sc-154) Abs (Santa Cruz Biotechnology, Dallas, USA). Phosphorylation of ERK and c-FOS in the whole cell lysate of GEN2.2 cells was analyzed by Western blotting using phospho-c-FOS-T325 Ab from Abcam (Cambridge, UK) and ERK Ab T202/Y204 (Santa Cruz Biotechnology, Dallas, USA) as described previously (15). After incubation with the appropriate horseradish peroxidase-conjugated secondary antibody, the membranes were washed and the protein bands were detected with Super Signal™ enhanced chemoluminiscent substrate detection reagent (ThermoFisher Scientific, Villebon-sur-Yvette, France). Densitometric analyses were performed using Amersham Imager 600 (GE Healthcare Life Science). Band intensities were normalized to GAPDH or Ponceau red.

### Determination of c-FOS by Dynamic Flow Cytometry

To determine total c-FOS by dynamic flow cytometry, 10^6^ GEN2.2 cells or 2 × 10^6^ PBMCs per milliliter were kept in the RPMI 1640 medium supplemented with 10% FCS. Aliquots of 10^6^ GEN2.2 cells or 8 × 10^6^ PBMCs were stimulated with 4 µg/ml CpG-A, 100 ng/ml PMA, 10 µg/ml of BDCA-2 mAb for 16 hr (GEN2.2 cells) or 4 hr PBMCs. Live/Dead cell discrimination was performed by Zombie Green™ Fixable Viability Kit (BioLegend, San Diego, USA). For flow cytometry analysis of total c-FOS, cells were fixed in 4% formaldehyde for 10 min, permeabilized by 90% methanol for 30 min, and stained by PE conjugated c-FOS (9F6) rabbit mAb (Cell Signaling, Danvers, USA). For determination of c-FOS in primary pDCs, PBMCs were stained by APC-conjugated anti-human CD123 mouse mAb (BD Biosciences, San Jose, USA) and FITC-conjugated anti-human lineage cocktail mouse Abs (BioLegend, San Diego, USA). pDCs in PBMCs population were defined as Lin-, CD123+ cell population. Samples were analyzed using a BD LSR FORTESSA cytometer (BD Biosciences, San Jose, USA) and data were processed using FLOWJO software (Treestar, San Carlos, USA).

### Cell Cycle Analysis

For analysis of cell cycle, 10^6^ GEN2.2 cells/ml of RPMI 1640 medium supplemented with 10% FCS were aliquoted in 1 ml quantities in 6-well flat-bottom culture plates and exposed to 1 µM PD0325901, 4 µg/ml CpG-A, and 10 µg/ml of BDCA-2 mAb for 16 h. The cells were then resuspended in the RPMI 1640 medium containing 6 µg/ml Hoechst 33342 Dye (ThermoFischer Scientific) and incubated at 37°C in 5% CO_2_ for 30 min and the amount of DNA was determined by flow cytometry. Live/Dead cell discrimination was performed by Zombie Green™ Fixable Viability Kit (BioLegend, San Diego, USA). Samples were analyzed using a BD LSR FORTESSA cytometer (BD Biosciences, San Jose, USA) and data were processed using FLOWJO software (Treestar, San Carlos, USA). Phases of the cell cycle were calculated by Dean-Jett-Fox model.

### Determination of Secreted IFN-α, TNF-α, and IL-6

The quantities of total IFN-α, TNF-α, and IL-6 produced by pDCs or GEN2.2 were measured in cell-free supernatants using human ELISA kits (IFN-α and IL-6 from Mabtech, and TNF-α from BD Biosciences). The index of synergism was determined from the following formula: the level of cytokine production after stimulation with the combination of CpG and PD0325901 divided by the sum of cytokine production level after stimulation with CpG and PD0325901 separately. PD0325901 alone did not induce a detectable quantity of respective cytokines. Combinations resulting in an index of synergism >1.5 were considered to be synergistic. The combinations resulting in an index of synergism ≤1.5 and in a 30% increase in stimulation compared to the stimulation observed with either of the two stimulators were considered to be additive.

### Statistical Analysis

Quantitative variables are expressed as the mean ± SEM (standard error of the mean). To compare the levels of cytokine production and transcription of c-FOS mRNA by pDCs, we used a Mann–Whitney or a Wilcoxon two-tailed non-parametric tests. For flow cytometry analyses, we used two-tailed *t*-test. Data were analyzed with GraphPad Prism 4 (GraphPad Software, La Jolla, CA). A *p* value ≤ 0.05 was considered to be significant.

## Ethics Statement

Peripheral blood mononuclear cells (PBMCs) from healthy anonymous donors were obtained from the national blood services (Etablissement Francais du Sang, Marseille, France). Blood samples were obtained after written consent following the approval of the EFS, Marseille, France and the Centre de Recherche en Cancérologie de Marseille (CRCM) in accordance to the convention signed the 20th May 2014.

## Author Contributions

Contribution: VJ, BA, and AF-H equally performed research, designed research, and analyzed data. TH, KT, and JW performed research and analyzed data. JN, DO, and PD designed research and analyzed data. LC and JP provided essential materials. RS and IH designed research, analyzed data, and wrote the paper.

## Conflict of Interest Statement

The authors declare that this research was conducted in the absence of any commercial or financial relationships that could be construed as a potential conflict of interest.

## Abbreviations

AP-1, activator protein 1; BLNK, B-cell linker protein; BCR, B cell receptor; BDCA-2, blood dendritic cell antigen 2; BST2, bone marrow stromal cell antigen 2; BTK, Bruton’s tyrosine kinase; FcRs, Fc receptors; geq, genome equivalent; HCV, hepatitis C virus; ITAM, immunoreceptor tyrosine-based activation motif; IL-1, interleukin 1; IFN-I α/β/ω, type I interferons; PMA, phorbol myristoyl acetate; PLCγ2, phospholipase Cγ 2; PKC, protein kinase C; RRs, regulatory receptors; SRE, serum response element; SYK, spleen tyrosine kinase; TNF-α, tumor necrosis factor α; TPL-2, tumor progression locus 2; TBK1, TANK binding kinase 1.
