# Supplementary material for: The MEK1/2-ERK Pathway Inhibits Type I IFN Production in Plasmacytoid Dendritic Cells
Source: Front Immunol. 2018 Feb 26;9:364. doi: 10.3389/fimmu.2018.00364 (PMC5835309; doi:10.3389/fimmu.2018.00364)
Supplement: Supplementary file 1 [file Presentation_1.PDF]

Supplementary Material

The MEK1/2-ERK pathway inhibits type I IFN production in plasmacytoid dendritic cells

Vaclav Janovec, Besma Aouar, Albert Font-Haro, Tomas Hofman, Katerina Trejbalova, Jan Weber, Laurence Chaperot, Joel Plumas, Daniel Olive, Patrice Dubreuil, Jacques A. Nunès, Ruzena Stranska*, and Ivan Hirsch*

*** Correspondence:** Ruzena Stranska, and Ivan Hirsch
ruzena.stranska@kuleuven.be, ivan.hirsch@inserm.fr

Supplementary Figures

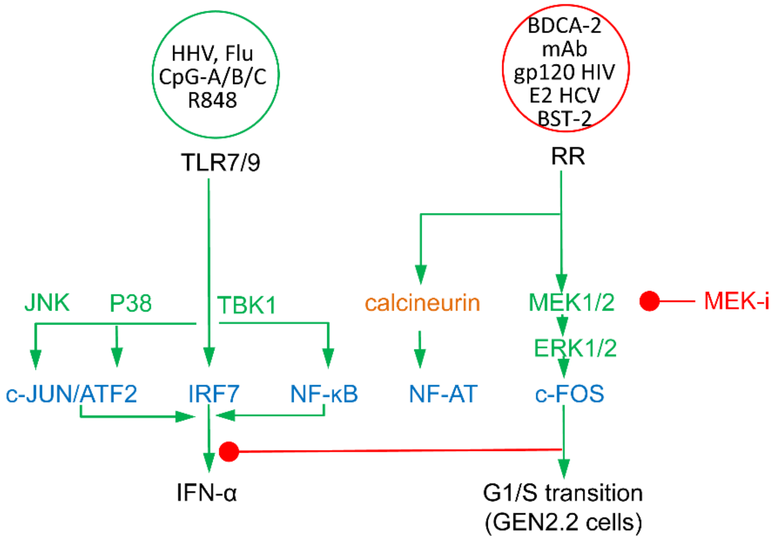

FIGURE S1 | TLR7/9 and BCR-like (RR-triggered) signaling pathways in pDCs. Protein kinases JNK, p38 MAPK, TBK1, MEK1/2 (in green), phosphatase calcineurin (in ochre), and transcription factor NF-κB (in blue) studied in this work are depicted. Transcription factors involved in regulation of expression of IFN-α are shown in blue. Positive signaling pathways are shown by green arrows, negative signaling is in red. IFN-α stimulating TLR7/9 agonists Human herpesviruses (HHV), influenza virus (Flu), CpG-A/B/C, R848 are grouped in a green circle. Agonists of RR (BDCA-2 mAb, gp120 HIV, E2 HCV, BST-2) are grouped in a red circle.

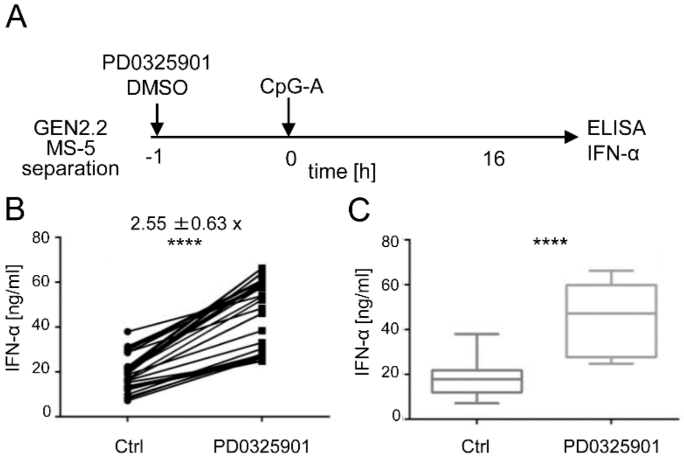

FIGURE S2 | Effect of MEK1/2 inhibitor PD0325901 on the production of IFN-α in CpG-A-stimulated GEN2.2 cells. (A) Experimental outline. GEN2.2 cells separated from MS-5 feeder cells were incubated or not with 1 µM PD0325901 for 1 h before stimulation with CpG-A. After a 16 h culture, the IFN-α production was determined in the cell-free supernatants by ELISA. **(B)** The data are shown as an aligned dot plot or **(C)** a box-and-whiskers plot (median [interquartile range (IQR)] 17.9 IQR [12.0-21.9] IFN-α (ng/ml), ctrl without PD0325901, 47.2 IQR [27.7-59,8] IFN-α (ng/ml) with PD0325901. N=34, ****, *p* <0.0001, two-tailed paired Wilcoxon test.

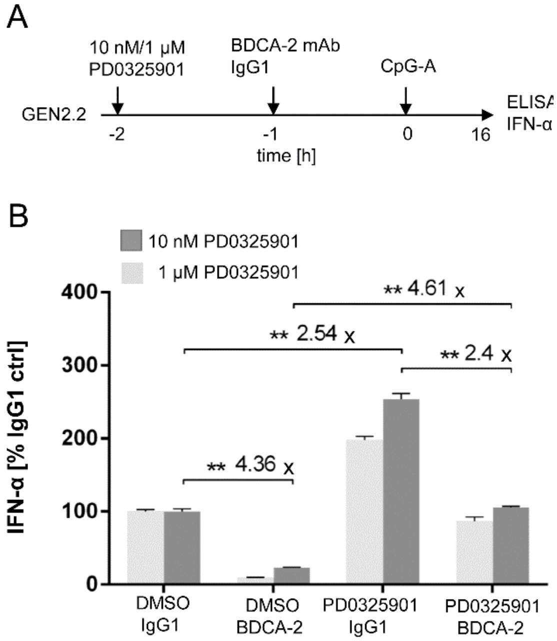

FIGURE S3 | Effect of MEK1/2 inhibitor PD0325901 on the blockade of IFN-α production by ligation of RRs of GEN2.2 cells with BDCA-2 mAb. (A) Experimental outline. GEN2.2 cells separated from MS-5 feeder cells were incubated with 10nM or 1 µM PD0325901 for 1 h before stimulation with BDCA-2 mAb and CpG-A. After a 16 h culture, the IFN-α production was determined in the cell-free supernatants by ELISA. **(B)** The IFN-α production was normalized to the level induced in pDCs by CpG-A in the presence of IgG1 and in the absence of the MEK1/2 inhibitor. The data show mean ±SEM of six independent experiments with GEN2.2 cells, **, *p* <0.01 two-tailed Mann-Whitney test.

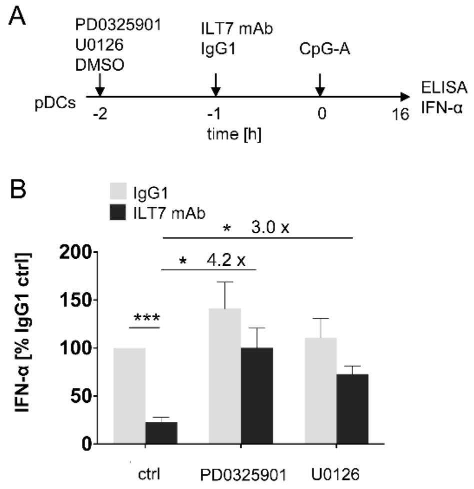

FIGURE S4 | Effect of MEK1/2 inhibitors PD0325901 and U0126 on the blockade of IFN-α production in primary pDCs by ligation of RR ILT7. (A) Experimental outline. Primary pDCs isolated from PBMCs of healthy donors were incubated with 1 μM U0126 (N=5) or 1 μM PD0325901 (N=6) for 1 h before stimulation with ILT7 antibodies and CpG-A. After a 16 h culture, the IFN-α production was determined in the cell-free supernatants by ELISA. **(B)** The data show mean ±SEM of IFN-α production in five independent experiments with U0126 and six independent experiments with PD0325901 normalized to the level of IFN-α induced in pDCs by CpG-A in the presence of IgG1 and in the absence of the MEK1/2 inhibitor. *, p <0.05; *** p <0.001; two-tailed paired Wilcoxon test.

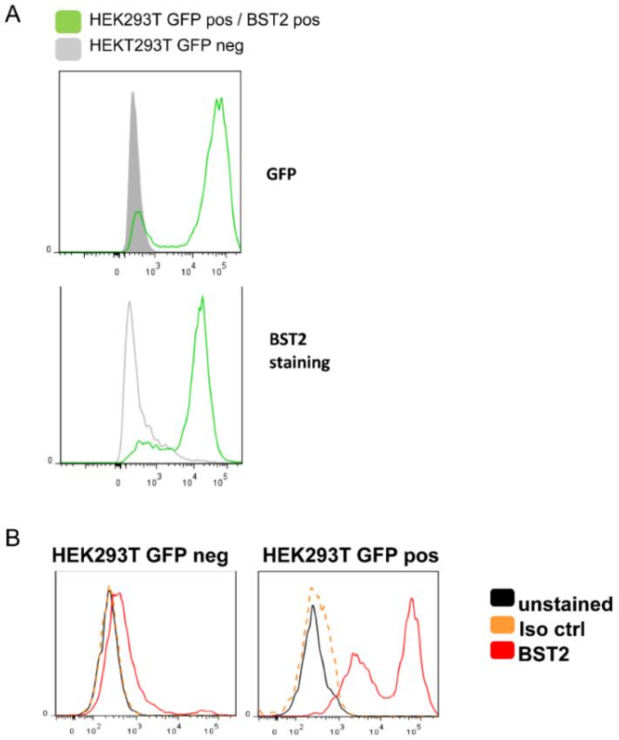

FIGURE S5 | Characteristics of BST2 expressing HEK293T cells. HEK293T cells transduced by lentivirus vector pRRL-BST2-GFP and sorted by GFP marker were analyzed for the expression of GFP and BST2 just after cell sorting **(A)** or after a 2-week culture **(B)**. Both the BST2-transduced (GFP+) cells and mock-transduced HEK293T cells are shown.

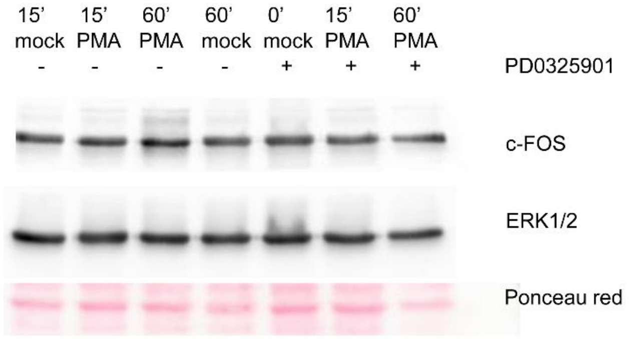

FIGURE S6 | c-FOS and ERK1/2 in PMA-stimulated GEN2.2 cells. GEN2.2 cells separated from MS-5 feeder cells and starved in a serum-free medium for 16 h were pretreated or not with MEK1/2 inhibitor PD0325901 for 1 h and then stimulated with PMA for 0, 15 and 60 min. c-FOS and ERK1/2 were analyzed using Western blotting with rabbit polyclonal c-FOS (sc-52) and ERK1/2 (sc-154) Abs.

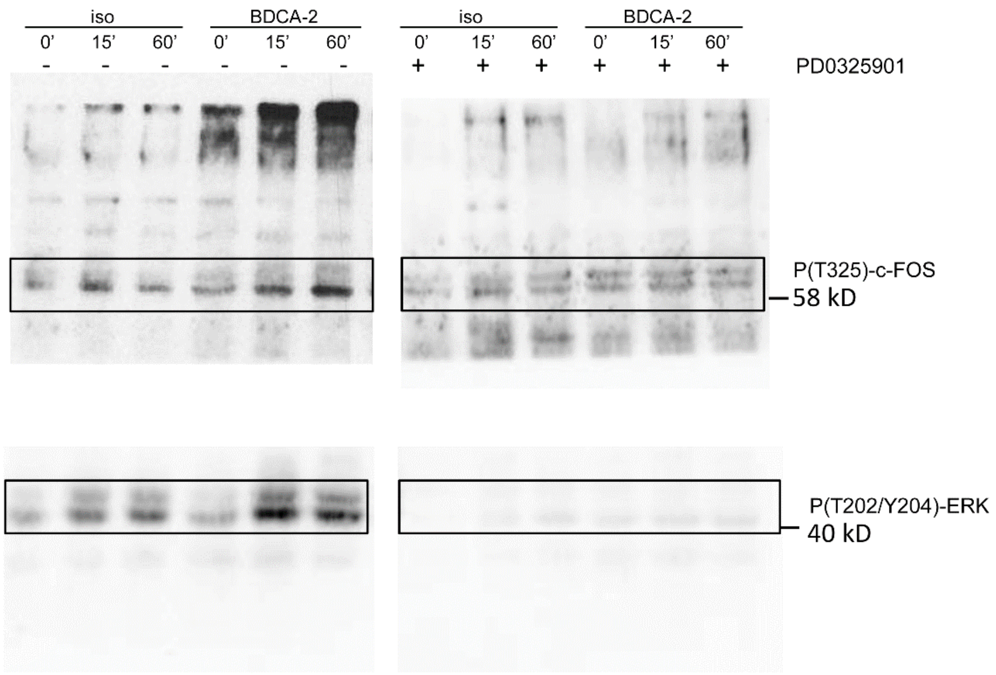

FIGURE S7 | Activation of c-FOS (A) and ERK (B) in GEN2.2 cells stimulated with BDCA-2 mAb – full scans of original gels. GEN2.2 cells separated from MS-5 feeder cells and starved in a serum-free medium for 16 h were pretreated or not with MEK1/2 inhibitor PD0325901 for 1 h and then stimulated with BDCA-2 mAb. **(A)** The activation of c-FOS was evaluated by analysis of c-FOS phosphorylation using Western blotting with the P(T325)-c-FOS antibody. **(B)** The phosphorylation of ERK-1/2 was determined by P(T202/Y204) ERK-1/2. Framed sections of the Western blot are shown in **Figure 10C**.
